# Composition of the CD27+ Memory-B-Cell Compartment Delineates Immunoglobulin Deficiency Endotypes

**DOI:** 10.21203/rs.3.rs-3838482/v1

**Published:** 2024-01-09

**Authors:** Oliva Starich, Jared M. Rieck, Wyatt J. Tarter, Camille J Hochheimer, Vijaya Knight, Jordan K Abbott

**Affiliations:** University of Colorado School of Medicine; Colorado School of Public Health; Colorado School of Public Health; Colorado School of Public Health; University of Colorado School of Medicine; University of Colorado School of Medicine

## Abstract

**Abstract Purpose:**

The finding of reduced numbers of class-switched memory B cells (CSM) in peripheral blood is widely used to assist the diagnosis and subclassification of CVID. Limited data exists on this finding in relation to the entire class of PADs. In this study, consecutive 8-marker comprehensive B-cell panel results were analyzed to determine how reduced CSM quantities might inform the pathophysiology of CVID and other humoral immunodeficiencies.

**Methods:**

Subpopulations of CD27+ memory B cells from 64 consecutive subjects with or without humoral immunodeficiency were examined to identify associations with diagnosis and serum immunoglobulin level. Results: CD27+IgM-IgD- percentage (CSM%) was correlated with IgG level in a discontinuous manner with an estimated change point of 9.7% (95% CI: 4.7, 12.4). All subjects with a CSM% below 9.7% had substantially lower serum IgG and IgA levels compared with those above 9.7. CSM% below 9.7% is not associated with serum IgM level. Rather, the proportion of CD27+IgMonly B cells (IgMonly or IgMonly%) is correlated with serum IgM.

**Conclusion:**

Low CSM% may mark an endotype of humoral immune dysfunction defined by either loss of class switching or critical failure of the coordinated production of both memory cells and long-lived plasma cells responsible for adequate immunoglobulin levels in humans. In patients with low CSM%, maintenance or expansion of IgMonly cells and IgM production suggests the former explanation, while concomitant loss of IgMonly cells suggests the latter. These findings provide a simple endotypic stratification method for future studies on the failed coordinated B cell response in humans with PAD.

## INTRODUCTION

In humans, most memory B cells are marked by the surface expression of CD27. These CD27^+^ memory B cells are typically divided based on surface expression of immunoglobulin isotypes into 4 CD27^+^ memory B cell subtypes: IgM^+^IgD^+^ (natural effector), IgM^−^IgD^−^ (class switched, CSM), IgM^+^IgD^−^ (IgMonly), and IgM^−^IgD^+^ (IgDonly) [1]. Natural-effector B cells resemble splenic marginal zone cells: they have divided less and carry less signs of B-cell receptor (BCR) selection than their memory counterparts [1–3]. These cells likely arise and function independent of T cell help, as CD40L-deficient patients partially preserve the natural effector population in the peripheral blood [4]. The class-switched B cell population carries the greatest number of cell divisions and the most evidence of BCR selection, indicating that most are the product of one or a series of germinal center reactions. The IgMonly population carries less evidence of cell division than the class-switched population but shows some evidence of selection leading to the conclusion that they arise from isolated primary germinal center reactions [1]. Finally, IgDonly cells show evidence of multiple divisions and carry highly mutated BCRs that are frequently autoreactive, leading to the conclusion that their relative anergy is the result of a regulatory mechanism [5]. Thus, the CD27^+^ B cell compartment can be divided into a T-cell-independent ontogeny and 3 T-cell-dependent groups separated by their extent of participation in germinal center reactions and the degree of BCR self-reactivity.

Following the initial characterization of CD27 on human B cells, studies sought to assign clinical significance to perturbations of this marker, particularly with regards to the classification and diagnosis of CVID. Brouet et al. first noted that the percentage of CD27^+^ B cells was highly variable in a small cohort of CVID patients; however, disproportionately fewer of CVID patients’ IgG- or IgA-expressing B cells expressed CD27 when compared to healthy controls [6]. Evaluating CVID patients, Jacquot et al. identified reductions in total CD27^+^ B cells, and Warnatz et al. identified significant reductions in CD27^+^IgD^−^ B cells, specifically, leading the latter group to propose low numbers of CD27^+^IgD^−^ B cells as a percentage of peripheral blood leukocytes as a marker of a subgroup of CVID [7, 8]. This classification was extended by Piqueras et al. who highlighted a distinction between CVID patients with both low CD27^+^IgD^−^ B cells and low CD27^+^IgD^+^ B cells and those with isolated reductions in CD27^+^IgD^−^ B cells [9]. Ultimately, these studies led to the suggestion that low CD27^+^IgD^−^ B cell numbers could be a useful supportive criterion for making the CVID diagnosis and were incorporated into the ESID working definition of CVID [10, 11].

Despite the generally accepted clinical utility of measuring CD27^+^IgD^−^ B cells, it has never been determined to what degree class-switched memory (CSM) B cell proportion is informative about the underlying humoral immune process. In this retrospective study, we analyzed consecutive 8-marker comprehensive B-cell panel results to determine if reduction in the representation of class-switched memory B cells – CD27^+^IgM^−^IgD^−^ in this study – informs the pathophysiology of immunoglobulin deficiency. First, we examine the relationship between CSM% as a proportion of total CD27^+^ memory cells and serum level of IgG, IgA, and IgM. Next, we examine the relationship between CSM% and the other CD27 + subtypes, natural effector and IgMonly B cells, and serum IgM level. Finally, we examine the relationship between CSM% and the diagnoses of CVID and other humoral immunodeficiency.

## METHODS

### Patient Population

This study was granted exempt status by the Colorado Multiple Institutional Review Board. Patients were identified for inclusion from a sample of 92 serially acquired B-cell panels (CBPs) drawn by the Allergy & Immunology Department at Children’s Hospital of Colorado between January 1, 2020, and December 31, 2020. Inclusion criteria included: age ≥ 5 years and absolute CD19 B-cell measurement within 60 days of B-cell panel results. Exclusion criteria included: use of rituximab and/or daratumumab in the 12 months preceding the B-cell panel date. Inclusion criteria were met by 67 patients; of those, 3 were excluded for daratumumab/rituximab use, resulting in a final sample size of 64 patients (n = 64) and 81 B-cell panel results.

All subjects included were assigned a diagnosis of CVID based on ICON criteria or by expert opinion when the workup was incomplete, such as in cases with borderline serum immunoglobulin levels or where polysaccharide antibody responses were not available [12]. In a descriptive analysis, those subjects were considered to have CVID if all other criteria were met. Patients that did not meet CVID criteria but were considered to have a humoral immune problem based on expert review were separately categorized. Study data were collected and managed using REDCap electronic data capture tools hosted at University of Colorado Denver – Anschutz Medical Campus [13].

### Flow Cytometry

Peripheral blood mononuclear cells (PBMC) were isolated from whole blood samples using density gradient centrifugation. PBMC viability was assessed by trypan blue exclusion and cells were resuspended in phosphate buffered saline (PBS) at 20×10^6^/mL. PBMC (100 mcL of the suspension) were added to a Duraclone IM B cell 8-color antibody panel tube containing the following stabilized antibodies: (CD45-KrO (Krome Orange), CD19-ECD (Electron Coupled Dye), IgD-FITC (Fluorescein isothiocyanate), CD27-PC7 (Phycoerythrin-cyanine 7), CD21-PE (Phycoerythrin), CD24-APC (Allophycocyanin), CD38-APC-A750 (Allophycocyanin-750), and IgM-PB (Pacific Blue) (Beckman Coulter, San Jose, CA). Following a 20-minute incubation at room temperature, protected from light, the cells were washed with PBS, fixed and data acquired on a Navios 10-color flow cytometer (Beckman Coulter). Data were analyzed using Kaluza Analysis Software, version 2.1 (Beckman Coulter).

### Definition of Class-Switched B cells

CSM B cells were defined as the percentage of CD19^+^CD27^+^ B cells that simultaneously lacked both surface IgD and surface IgM (CD19^+^CD27^+^IgM^−^IgD^−^ memory B cells). This value was chosen instead of CD27^+^IgD^−^ B cells to account for IgMonly memory cells that express surface IgM but not IgD. Absolute B cell quantification is not performed during the B-cell flow cytometry assay, so as a surrogate of absolute numbers of B the absolute number of CSM cells was calculated as the product of the proportion of total CD19^+^ B (the grandparent gate) and the absolute CD19 count from a recent TBNK flow cytometry assay.

### Statistical Analyses

Descriptive statistics (counts and percentages for categorical variables, means and standard deviation or medians and mins/maxes for continuous) were calculated for demographic variables. Multinomial logistic regression was performed to model the relationship of CVID diagnosis (diagnostic categories of confirmed CVID diagnosis (Yes), incomplete workup but likely CVID (Maybe), and No CVID) with CD27^+^IGM^−^IGD^−^ CSM count/percentage, and binary logistic regression was performed to model the relationship of any immunodeficiency diagnosis with CSM count/percentage. Sensitivity analyses included using the average of repeat panels among the 15 individuals with more than one panel to examine if outlier panels impacted the results, along with a GEE approach for the logistic and linear regressions to assess the effects of within-subject correlation in repeat panels.

Preliminary data analysis revealed a non-linear relationship between IgG and CSM percentage. A Bayesian changepoint analysis was conducted using the mcp R package to determine if there was a change-point in the relationship of IgG with CSM percentage. A disjoined slope model, an intercept only model, and a varying slope model were compared using Bayes factors to determine the most appropriate model. All models were fit using 3 chains with 3000 iterations each, using a burn in period of 500 iterations. We fit models using both a disjoined slope and varying slope, but report results from the disjoined slope model, which achieved a superior model fit. After selecting a final changepoint model, hypothesis testing was performed to determine if the observed changepoint was below the threshold of 14.1% previously established by the Children’s Hospital Immunodiagnostic Laboratory. The mean change from the selected changepoint model was used to dichotomize CSM% based on whether a subject’s values fell above or below this mean value. This dichotomous variable was then used in Tobit regressions to model the relationship between IgA or IgM and low vs not low CD27^+^IGM^−^IGD^−^ CSM percentage. The relationships between IgM and the percentage IgMonly B cells and the percentage of natural effector B cells both separately and together were also modeled via Tobit regression.

All models were adjusted for sex and an alpha of 0.05 was set as the significance level. Analyses were performed in R version 4.2.1 (Vienna, Austria).

## RESULTS

### Patient Characteristics

#### Demographics

A demographic summary of the cohort is presented in Table 1. The mean age was 15.4 and 32 (50%) were female sex. Most patients were white (75%), and 78.1% did not identify as Hispanic or Latino.

#### Diagnostic Categories

The ICON criteria recommend excluding other causes of hypogammaglobulinemia, including the following causes: drug-induced-, single gene and other defects, chromosomal anomalies, infectious diseases, malignancy, and other systemic disorders. After removing subjects according to this criterion, 10 subjects were felt to have CVID by either meeting criteria or by expert opinion when data was incomplete for assessing certain criteria. An additional 5 subjects would have otherwise been diagnosed with CVID if not for the exclusion criteria. Therefore, the number of subjects with CVID varies depending on how the exclusion criteria are interpreted. Thirty-one subjects had no humoral immune deficiency. The remaining subjects were categorized as having a non-CVID humoral immune problem (n = 23 with strict exclusion criteria).

#### Repeat Measures

Of the 64 patients included in the final analysis, 13 patients were linked to two separate CBPs measured at different times, and 2 patients were linked to three CBPs.

##### Clinical Reference Ranges May Not Be Stringent Enough for CD27 ^+^ IGM ^−^ IGD ^−^ CSM Deficiency.

**Figure 1:**
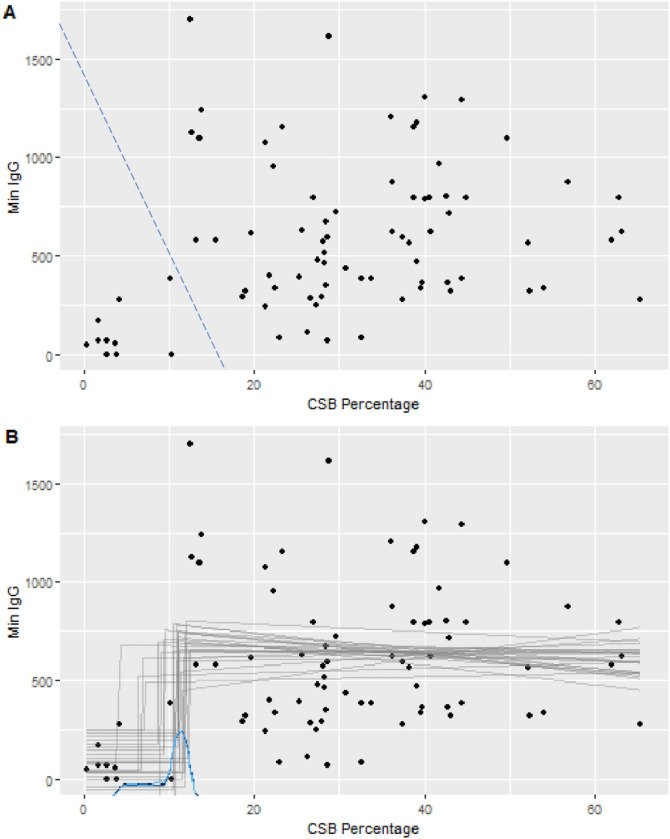
Relationship Between CSM% and serum IgG Level The CSM% of all B-cell phenotype panel results is plotted against the historical lowest serum IgG level (mg/dL). **A.** The blue dashed line divides the scatter plot in the area where a perceived transition occurs between high and low CSM%. **B.** The results of Bayesian change-point analysis for the population as a whole are shown with discontinuous plot estimates in grey and the distribution of changepoint estimates in blue. *MinIgG is the lowest available serum IgG level, preceding IgG replacement. CSB percentage is CSM% as indicated in the text*

In preliminary analyses, we observed a modest but potentially discontinuous relationship between IgG and CSM% (Fig. 1A). We hypothesized that a threshold existed below which severe IgG deficiency was more likely, and that the clinical threshold of 14.1% used in our local clinical laboratory was overestimating this breakpoint. To explore the possibility that a lower threshold more accurately captured pathological CD27^+^IgM^−^IgD^−^ CSM deficiency, threshold analysis was performed. A Bayesian changepoint analysis using a disjoined slope at the changepoint found a mean CSM% change-point of 9.7% with a 95% Credible Interval of 4.7% – 12.4% (Fig. 1B). To evaluate if the model supported the hypothesis that the changepoint was below the accepted clinical threshold, we calculated the proportion of Markov-Chain Monte Carlo (MCMC) samples that have a changepoint below 14.1% CSM. All posterior samples met this criterion (Bayesian p-value = 1), indicating strong support that in this sample that the observed change in the relationship between IgG and CSM % occurs below the established clinical threshold.

#### The Redefined CD27 ^+^ IGM ^−^ IGD ^−^ CSM cutoff is associated with serum IgA level.

Using the 9.7% change-point as a cutoff to dichotomize low (CSM-cp-low) versus normal-to-high CSM, we sought to determine if CSM-cp-low had different IgA and IgM compared to those with normal-to-high CSM. Tobit regression models revealed that the dichotomized CSM-cp-low is a significant predictor of serum IgA level but not IgM. IgA is lower by 194.35 (95% CI: 61.88, 326.82; p = 0.005) units in the CSM-cp-low group compared to those with normal-to-high CSM (Table 2).

#### IgM Levels are associated with percentage of CD27 ^+^ IgMonly and Not CD27 ^+^ IgM^+^ IgD ^+^ Natural Effector Cells

**Figure 2:**
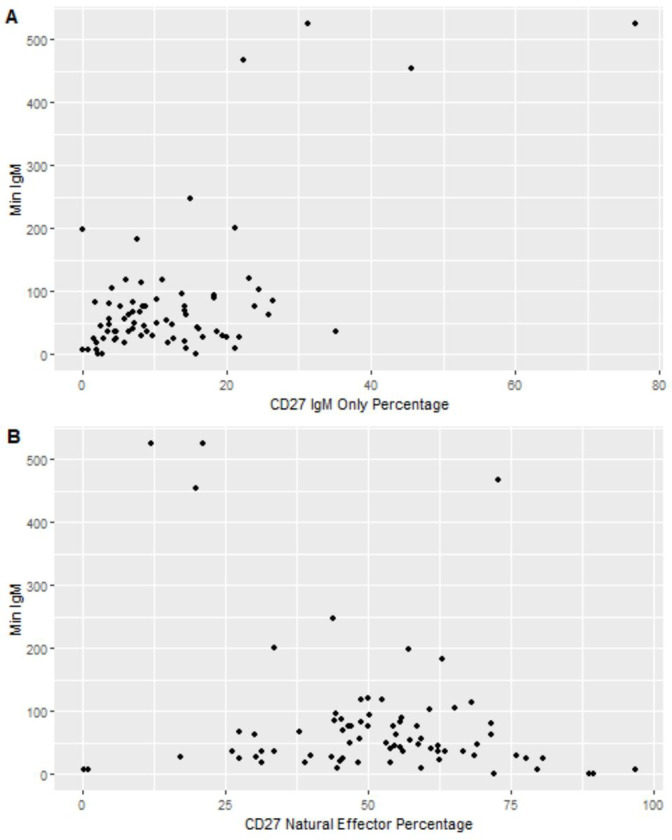
Relationship Between IgMonly or natural effector cells and serum IgM Level **A.** IgMonly % is plotted against the lowest available serum IgM level (mg/dL). **B.** Natural effector % is plotted against the lowest available serum IgM level (mg/dL). *For display purposes, for both A and B, left-truncated values of serum IgM are represented as zero*

Since CSM B cells do not secrete IgM, the finding that subjects from the CSM-cp-low group did not have lower serum IgM levels was as expected (Table 3); however, upon further inspection we found that 5 of 6 subjects (84%) in the CSM-cp-low group had low IgM levels – four of them having levels less than 10 mg/dl. The remaining subject had robust levels of IgM in the blood despite lacking CSM B cells entirely. Interestingly, the IgM only B cell population in this subject was expanded relative to the other subjects. Considering this finding, it was of interest to explore the associations of serum IgM levels with IgMonly percentage and the percentage of natural effector cells. Tobit regression models revealed that after adjusting for sex, IgM was higher by 6.3 units for each percentage increase in CD27IgMonly (95% CI: 4.6–8.0; p < 0.001) while IgM was lower by 1.65 units for each percentage increase in CD27 natural effector cells (95% CI: −3.05, −0.25; p = 0.021) (Fig. 2, Table 4,5). When controlling for the effects of CD27IgMonly percentage, the relationship between serum IgM and CD27 natural effector cells was no longer significant. These findings indicate that while low IgM levels are frequently found in patients with low CSM%, the IgMonly percentage takes precedence as a better predictor of serum IgM level.

#### Stability of Low CSM Percentage

Since low CSM% was associated with impaired IgG production, we reviewed our data for evidence of this being a stable finding over time. Three of 7 (43%) subjects with CSM% less than 9.7% had repeat panels performed. For those 3 subjects, CSM% remained less than 9.7% for a period of 308 days, 277 days, and 165 days. No subject with a CSM% above 9.7% had a repeat value below 9.7%. This suggests that low CSM% may be stable over the period of one year, but this hypothesis should be formally tested in future research.

#### Is the number or percentage of CD27 ^+^ IGM^−^ IGD ^−^ CSM cells associated with CVID?

Having found a relationship between low CSM% and low serum immunoglobulin levels, we next examined the relationship between CSM% and the diagnosis of CVID. Multinomial regression analysis found no significant association between the CVID categories and either percentage or absolute number of CSM B cells (p = 0.4 and 0.8, respectively) (Tables 6,7). In contrast, binary logistic regression found a significant relationship between CSM% and any humoral immunodeficiency (Table 8) but not between the absolute number of CSM B cells and any humoral immunodeficiency. Thus, CSM% is inversely associated with the odds of having a humoral immunodeficiency despite not being associated with the diagnosis of CVID.

## DISCUSSION

A fundamental feature of the CSM% that was revealed in this work is the existence of a threshold level below which severe hypogammaglobulinemia is almost certainly present. In our study, this cutoff was lower than what had been established in the Children’s Hospital of Colorado Immunology Laboratory from a cohort of healthy adults. Reference ranges for multiparameter flow cytometry assays are typically based on a small, local healthy population, and the cutoff for truly abnormal results is generally unknown. Here we show that at least for our local laboratory, the reported lower limit of normal may overestimate the number of patients with quantitative deficiency of the CSM population.

Perhaps the most surprising finding of this study is that low CSM B cells are associated not only with lower levels of serum IgG and IgA but are frequently seen with low serum IgM level. Serum IgG and IgA are largely the product of long-lived plasma cells in the bone marrow, a cell type that emerges following germinal center reactions in lymphoid organs [14]. In contrast, IgM-producing plasma cells are infrequently found in the bone marrow. In this study, we found that that the predominant factor associated with serum IgM level is the relative amount of IgMonly cells. IgMonly cells are typically a minor population in the blood with a comparable population found in the intestines [15]. These cells show evidence of antigen experience and selective pressure, both signs of germinal center involvement [5, 16]. It is therefore likely that the majority of IgM detected in the blood arises directly from IgM^−^producing plasma cells whose progenitors participated in a germinal center reaction. Thus, the combination of low CSM B cells with low serum IgG, IgA, and IgM implies a failure of their common thread, the germinal center reaction.

Considering our findings, we propose a novel classification system for humoral immune abnormality based on the presumed pathophysiologic dysfunction underlying a deficiency of CSM. In group A, both CSM and IgMonly proportions are significantly decreased in conjunction with reduction in all serum immunoglobulins. In this scenario, a presumptive defect in germinal center activity prevents the development of both cell types with a bottleneck limiting the formation of downstream long-lived plasma cells. In group B, CSM are reduced but CD27IgMonly are either at usual levels or are increased, and IgM levels in the blood persist above the reference lower limit. In this group, a general failure of germinal center activity is not required, as this scenario could arise if class-switching were defective. Alternatively, there could be a defect in germinal center activity that is overcome under certain circumstances such as infection or autoimmunity but primarily restricted to the production of IgM-producing plasma cells. These groups are presumed to exist independent of whether the individual is diagnosed with CVID or another humoral immunodeficiency. Group C would include patients with adequate representation of CSM cells, indicating maintenance of germinal center activity but failure to form robust long-lived plasma cells.

This study finds that having low CSM% is not exclusive to CVID, but it is associated with humoral immunodeficiency in general. This association makes sense given that germinal center failure is not exclusive to CVID. This finding could also be the result of ambiguity in the CVID diagnostic criteria used to classify the patients. In this cohort, 6 of 7 subjects with CVID and CSM% <14.1% would be excluded based the ICON exclusion criteria that excludes genetic diagnoses. When a more stringent CSM% cutoff of 9.7% is used, the low-CSM group goes from 6 subjects with CVID to 1 with CVID. In that scenario, one would conclude that low CSM% is not specific for the CVID diagnosis; however, if the subjects in this group were not excluded from the CVID diagnosis because of alternative genetic explanations, potentially one would reach the opposite conclusion. Rather than shedding light on the pathophysiology of CVID, this highlights an emerging difficulty with crafting CVID diagnostic criteria. While CVID has historically been a diagnosis of exclusion, excluding patients based on having a monogenic immune disorder or having received B-cell cytotoxic therapy many years prior may unnecessarily limit the number of qualifying research subjects. This problem is likely to worsen as previously unrecognized monogenetic etiologies of CVID-like disease are increasingly identified. Given this limitation it is difficult to determine whether low CSM% is specific for the CVID diagnosis. However, low CSM% is clearly associated with defective humoral immunity.

## CONCLUSIONS

In summary, we provide evidence for a reconsideration of how the finding of low class-switched B cells should be interpreted. Rather than a marker of CVID or a specific sign of defective class switching, it is possibly an indicator of severe problems with progression of B cells through the completion of the process that generates cells with the capacity to produce significant amounts of immunoglobulin. The clinical utility of such knowledge is twofold. First, it provides a window into the underlying pathology of the humoral immune dysfunction being investigated. Secondly, in the context of investigating hypogammaglobulinemia, it has the potential to help distinguish disrupted B-cell functional pathways from the problems of protein loss and hyper-catabolism of immunoglobulins. Thirdly, it provides a novel approach to stratification of humoral immune abnormalities, focusing more on pathophysiology than a collection of clinical attributes. Additional investigation stratifying by these criteria may reveal important prognostic information or associations with other clinical features and will be worth further investigation in the future as more extensive, longitudinal data become available.

## Figures and Tables

**Table 1.** Demographics of the Study Group

	Overall (n=64)
**Panel Age**	
Mean (SD)	15.4 (6.47)
Median [Min, Max]	14.6 [5.46, 39.8]
**Sex**	
Female	32 (50.0%)
Male	32 (50.0%)
**Race**	
American Indian or Alaska Native	1 (1.6%)
Asian	2 (3.1%)
Black or African American	1 (1.6%)
More than one Race	4 (6.3%)
Native Hawaiian or Other Pacific Islander	1 (1.6%)
Unknown or Not Reported	7 (10.9%)
White	48 (75.0%)
**Ethnicity**	
Hispanic or Latino	11 (17.2%)
Not Hispanic or Latino	50 (78.1%)
Unknown or Not Reported	3 (4.7%)

**Table 2:** Tobit Regression of IgA on Percentage of Class-Switched B-cells with Changepoint

	IgA		
*Predictors*	*Estimates*	*CI*	*p*
Sex [Male]	−41.69	−103.18–19.81	0.181
below CP	−194.35	−326.82 – −61.88	**0.005**
Observations	77. *below CP = group with CSM% < 9.7%.*		

**Table 3:** Tobit Regression of IgM on Percentage of Class-Switched B-cells with Changepoint

	IgM		
*Predictors*	*Estimates*	*CI*	*p*
Sex [Male]	−53.64	−105.60 – −1.68	**0.043**
below CP	−96.52	−207.03–13.98	0.086
Observations	77. *below CP = group with CSM% < 9.7%.*		

**Table 4:** Tobit Regression of IgM on Percentage of CD27 IgM Only Cells (CD27 + IgD-IgM+)

	IgM		
*Predictors*	*Estimates*	*CI*	*p*
CD27 IgM Only	6.30	4.60–8.00	**< 0.001**
Sex [Male]	−40.18	−79.24 – −1.13	**0.044**
Observations	77		

**Table 5:** Tobit Regression of Min IgM on Percentage of CD27 Natural Effector Cells (CD27 + IgD + IgM+)

	Min IgM		
*Predictors*	*Estimates*	*CI*	*p*
CD27 Natural Effector	−1.65	−3.05 – −0.25	**0.021**
Sex [Male]	−43.12	−93.24–7.00	0.091
Observations	77		

**Table 6:** Multinomial Regression of CVID Diagnosis on Percent CD27 Switched

Characteristic	Expert opinion CVID (“Maybe”)			Criteria CVID only (“Yes”)		
	OR	95% CI	p-value	OR	95% CI	p-value
Percent CD27 Switched	1.00	0.95, 1.05	> 0.9	0.97	0.91, 1.04	0.4
Male	3.04	0.57, 16.4	0.2	0.96	0.13, 7.30	> 0.9

**Table 7:** Multinomial Regression of CVID Diagnosis on Count CD27 Switched

Characteristic	Expert opinion CVID (“Maybe”)			Criteria CVID only (“Yes”)		
	OR	95% CI	p-value	OR	95% CI	p-value
Count CD27 Switched	1.00	0.96, 1.05	0.8	0.95	0.84, 1.06	0.4
Male	3.14	0.59, 16.9	0.2	1.08	0.14, 8.45	> 0.9

**Table 8:** Logistic Regression of Immunodeficiency Status on Percent of CD27switched

	Humoral Immunodeficiency		
*Predictors*	*Odds Ratios*	*CI*	*p*
CD27switched	0.96	0.93, 0.99	**0.026**
Sex [Male]	2.10	0.84, 5.37	0.116
Observations	81		
